# Lipoteichoic Acid of Probiotic *Lactobacillus plantarum* Attenuates Poly I:C-Induced IL-8 Production in Porcine Intestinal Epithelial Cells

**DOI:** 10.3389/fmicb.2017.01827

**Published:** 2017-09-21

**Authors:** Kyoung Whun Kim, Seok-Seong Kang, Sun-Je Woo, Ok-Jin Park, Ki Bum Ahn, Ki-Duk Song, Hak-Kyo Lee, Cheol-Heui Yun, Seung Hyun Han

**Affiliations:** ^1^Department of Oral Microbiology and Immunology, DRI, and BK21 Plus Program, School of Dentistry, Seoul National University Seoul, South Korea; ^2^Department of Agricultural Biotechnology and Research Institute for Agriculture and Life Sciences, Seoul National University Seoul, South Korea; ^3^Department of Food Science and Biotechnology, Dongguk University-Seoul Seoul, South Korea; ^4^Department of Animal Biotechnology, Chonbuk National University Jeonju, South Korea; ^5^Institute of Green Bio Science Technology, Seoul National University Seoul, South Korea

**Keywords:** probiotics, lipoteichoic acid, viral infection, inflammation, intestinal epithelial cells

## Abstract

Probiotics in livestock feed supplements are considered a replacement for antibiotics that enhance gastrointestinal immunity. Although bacterial cell wall components have been proposed to be associated with probiotic function, little evidence demonstrates that they are responsible for probiotic functions in livestock. The present study demonstrated that lipoteichoic acid (LTA) of *Lactobacillus plantarum* (Lp.LTA) confers anti-inflammatory responses in porcine intestinal epithelial cell line, IPEC-J2. A synthetic analog of viral double-stranded RNA, poly I:C, dose-dependently induced IL-8 production at the mRNA and protein levels in IPEC-J2 cells. Lp.LTA, but not lipoprotein or peptidoglycan from *L. plantarum*, exclusively suppressed poly I:C-induced IL-8 production. Compared with LTAs from other probiotic *Lactobacillus* strains including *L. delbrueckii*, *L. sakei*, and *L. rhamnosus* GG, Lp.LTA had higher potential to suppress poly I:C-induced IL-8 production. Dealanylated or deacylated Lp.LTA did not suppress poly I:C-induced IL-8 production, suggesting that D-alanine and lipid moieties in the Lp.LTA structure were responsible for the inhibition. Furthermore, Lp.LTA attenuated the phosphorylation of ERK and p38 kinase as well as the activation of NF-κB, resulting in decreased IL-8 production. Taken together, these results suggest that Lp.LTA acts as an effector molecule to inhibit viral pathogen-induced inflammatory responses in porcine intestinal epithelial cells.

## Introduction

Probiotic bacteria are viable and defined microorganisms, which alter the composition and function of the intestinal microflora of humans and animals (Schrezenmeir and de Vrese, 2001). A set of major criteria for probiotics includes lack of transmissible antibiotic resistance genes, persistence of viability in the gastrointestinal tract, experimentally and clinically proven health benefits and safety for human and animal use (Lievin-Le Moal and Servin, 2014). Through production of metabolites including organic acids and bacteriocins, probiotic bacteria have beneficial effects on the intestines of mammals (O’Shea et al., 2012). The most extensively studied and widely used probiotic bacteria are *Lactobacillus* and *Bifidobacterium* (Kopp-Hoolihan, 2001), which directly remove pathogens by producing antimicrobial compounds, decreasing pH by acids such as lactic acid, competing with pathogens for adhesion and colonization, as well as nutrients and other growth factors in the gut (Kailasapathy and Chin, 2000; Rolfe, 2000).

Increasing evidence suggests that probiotic bacteria regulate inflammation in a number of ways. For example, probiotic bacteria enhance the epithelial barrier function to prevent chronic inflammation in the gut (Bermudez-Brito et al., 2012). Certain probiotic strains inhibit pro-inflammatory cytokine-impaired epithelial integrity (Donato et al., 2010). By increasing the production of metabolites such as butyrate, probiotic bacteria suppress intestinal inflammatory responses (Thomas and Versalovic, 2010). Probiotic bacteria confer anti-inflammatory responses by modulating signaling pathways including nuclear factor (NF)-κB, mitogen-activated protein kinase (MAPK) and peroxisome proliferator-activated receptor gamma (PPARγ) (Thomas and Versalovic, 2010). In addition, probiotic lactobacilli down-regulate the production of interleukin (IL)-6 and IL-8 in intestinal epithelial cells (Villena and Kitazawa, 2014; Noh et al., 2015). Probiotic bacteria also exert inflammatory responses by interacting with Toll-like receptors (TLRs). TLR2 can be particularly important for regulating inflammatory signaling pathways for Gram-positive bacteria (Noh et al., 2015).

Although probiotic bacteria have a positive impact on certain infections, inflammatory reactions, and allergic diseases, clinically employed probiotics have been revealed as species-and strain-specific (Snel et al., 2011; Agustina et al., 2012). Several reports suggest that individual probiotic effector molecules communicating with hosts may underlie probiotic effects (Bron et al., 2013; Lee et al., 2013). Several effector molecules such as peptidoglycan (PGN) and lipoteichoic acid (LTA) are the major cell wall components of Gram-positive bacteria that can be considered the pivotal components for immunomodulating effects (Lee et al., 2013). In particular, the D-alanine moiety of LTA structure is considered an important functional group for immunostimulating effects (Morath et al., 2001). Moreover, lipid moieties of the LTA structure are also considered to be immunomodulating groups (Morath et al., 2002). LTA from pathogenic bacteria is responsible for the initiation and development of inflammatory responses (Kang et al., 2016). In contrast, *Lactobacillus* LTA is involved in anti-inflammatory responses (Grangette et al., 2005; Noh et al., 2015). Moreover, PGN derived from lactobacilli reduces inflammatory responses in the colon (Macho Fernandez et al., 2011), suggesting that the cell wall of probiotic lactobacilli contains potential immunomodulators.

Antibiotics have been used to prevent diarrhea and promote growth in farm animals including pigs (Thacker, 2013). However, because of increased negative effects including emerging bacterial resistance to antibiotics (van der Fels-Klerx et al., 2011), probiotic lactobacilli are suggested as an alternative strategy in swine production. Beneficial effects of probiotic lactobacilli such as regulation of intestinal microflora, prevention of intestinal pathogens and maintenance of intestinal barrier function (Yang et al., 2015) are suggested. Nevertheless, the cell wall-associated molecules of probiotic lactobacilli that attenuate gastrointestinal inflammatory reactivity have not yet been fully elucidated. In this study, we investigated the effects of probiotic *Lactobacillus plantarum* LTA to reduce IL-8 production in porcine intestinal epithelial cells.

## Materials and Methods

### Bacteria and Reagents

*L. plantarum* K8 (KCTC 10887BP) was obtained from the Korean Collection for Type Culture (Daejeon, South Korea). Poly I:C was purchased from InvivoGen (San Diego, CA, United States). Pam2CSK4 was purchased from EMC Microcollection GmbH (Tübingen, Germany). Antibodies specific to p38 kinase, phospho-p38 kinase, ERK, phospho-ERK, IκBα, and β-actin were obtained from Cell Signaling Technology (Beverly, MA, United States).

### Preparation of Cell Wall Components of *Lactobacillus*

*L. plantarum* LTA (Lp.LTA) was prepared and the absence of unwanted biological contaminants such as endotoxins, nucleic acids, and proteins was examined as described previously (Ryu et al., 2009). D-Alanine-deficient Lp.LTA (dealanylated) and acyl chain-deficient structure of Lp.LTA (deacylated) were prepared as described previously (Noh et al., 2015). LTAs from *L. delbrueckii*, *L. sakei*, and *L. rhamnosus* GG were kindly provided from Prof. Dae Kyun Chung (Kyung Hee University, Suwon, South Korea). Lipoproteins of *L. plantarum* were isolated as described previously (Kang et al., 2015). PGN from *L. plantarum* was prepared as described previously (Noh et al., 2015).

### Cell Culture

A non-transformed IPEC-J2 cell line, originally isolated from jejunum of a neonatal piglet, was obtained from the American Type Culture Collection (Manassas, VA, United States), and grown in Dulbecco’s Modified Eagle’s Medium and Ham’s F-12 media (1:1) (HyClone, Logan, UT, United States) supplemented with 10% fetal bovine serum (Gibco, Burlington, ON, Canada), 1% insulin-transferrin-selenium G supplement (Gibco), 100 U/ml penicillin, and 100 μg/ml streptomycin (HyClone) at 37°C in a 5% CO_2_ atmosphere in a humidified incubator.

### Real-time Quantitative PCR

After simultaneous treatment with indicated stimuli in IPEC-J2 cells for 3 h, total RNA was extracted using TRIzol reagent (Invitrogen, Carlsbad, CA, United States) according to the manufacturer’s instructions. Five micrograms of total RNA was subjected to complementary DNA (cDNA) synthesis using random hexamers and reverse transcriptase (Promega, Madison, WI, United States). Real-time quantitative PCR was performed with SYBR Premix II Ex Taq (Takara Bio, Shiga, Japan) using an ABI Prism 7500 Sequence Detection System (Applied Biosystems, Foster City, CA, United States). Amplification of cDNA was performed as follows: denaturation for 10 s at 95°C and amplification for 40 cycles of 5 s at 95°C and for 34 s at 60°C. The ΔCt was obtained by subtracting the Ct value of β-actin gene from the Ct value of IL-8 gene and ΔCt of the non-treatment control group was used as the calibrator. The fold change was calculated by 2^-ΔΔCt^, where ΔΔCt was the difference between ΔCt and the ΔCt calibrator value, which was assigned a value of 1 arbitrary unit. Sequences of specific primers for IL-8 and β-actin were as follows: IL-8 sense primer, 5′-CCTGCTTTCTGCAGCTCTCT-3′; IL-8 anti-sense primer, 5′-CAGTGGGGTCCACTCTCAAT-3′; β-actin sense primer, 5′-TG TTCGAGACCTTCAACACG-3′; β-actin anti-sense primer, 5′-ATCCCCAGAGTCCATGACAA-3′.

### Enzyme-Linked Immunosorbent Assay (ELISA)

IPEC-J2 cells were seeded in 96-well culture plates and grown until fully confluent. After simultaneous treatment with indicated stimuli for 24 h, the culture media were collected and IL-8 was determined using a porcine IL-8 ELISA kit (R&D Systems, Minneapolis, MN, United States) according to the manufacturer’s instruction.

### Western Blot

IPEC-J2 cells were seeded in 6-well culture plates and grown until fully confluent. After simultaneous treatment with indicated stimuli for 30 min, the cells were lysed in a lysis buffer. Equal amounts of protein were resolved on 10% SDS-PAGE gels and electro-transferred onto PVDF membranes (Millipore, Bedford, MA, United States). After blocking with 5% bovine serum albumin, membranes were probed with primary antibodies specific for p38 kinase, phospho-p38 kinase, ERK, phospho-ERK, IκBα, or β-actin (Cell Signaling Technology). After washing, membranes were incubated with HRP-conjugated anti-rabbit IgG as secondary antibody. Immunoreactive bands were detected using a luminescent image analyzer (Fuji Film, Tokyo, Japan).

### Statistical Analysis

Statistical significance was measured using Student’s *t*-test and differences were considered significant when *P* < 0.05 by comparing the experimental group with appropriate control. The data represented one of three independent experiments unless otherwise stated.

## Results

### Poly I:C Induces IL-8 Production in IPEC-J2 Cells

**FIGURE 1:**
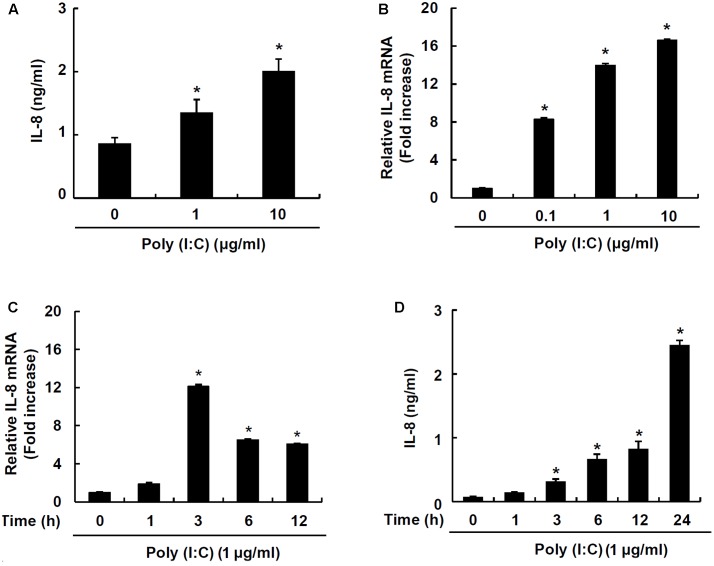
TLR3 ligand poly I:C induces IL-8 production in IPEC-J2 cells. IPEC-J2 cells (5 × 10^5^ cells/ml) were plated in 96-well plates and treated with 1 or 10 μg/ml poly I:C **(A)** for 24 h in serum-free medium. Then, the supernatants were collected and IL-8 secretion was determined by ELISA. IPEC-J2 cells (5 × 10^5^ cells/ml) were plated in 12-well plates and treated with 0.1, 1, or 10 μg/ml poly I:C for 3 h **(B)**, or 1 μg/ml poly I:C for 0, 1, 3, 6, or 12 h in serum-free medium **(C)**. After treatment, total RNA was extracted and IL-8 mRNA expression was determined by real-time PCR. Data are mean ± standard deviation of triplicate samples. **(D)** IPEC-J2 cells (5 × 10^5^ cells/ml) were plated in 96-well culture plates and treated with 1 μg/ml poly I:C for 1, 3, 6, 12 and 24 h, and IL-8 secretion was determined by ELISA. Data are expressed as mean value ± standard deviation of triplicate samples. Asterisk (^∗^) indicates significant difference compared with the non-treatment control group (*P* < 0.05).

IPEC-J2 has been characterized and commonly used as an *in vitro* model for studying porcine intestinal pathogen–host interactions (Botic et al., 2007). To examine whether a synthetic analog of viral double-stranded RNA, poly I:C, induces chemokine production, IPEC-J2 cells were stimulated with poly I:C. Poly I:C significantly increased IL-8 secretion at 1 and 10 μg/ml poly I:C (**Figure 1A**). Next, mRNA expression of IL-8 was further examined in IPEC-J2 cells stimulated with 0.1, 1, or 10 μg/ml poly I:C for 3 h. IL-8 mRNA expression was significantly up-regulated at 0.1 μg/ml up to 10 μg/ml poly I:C (**Figure 1B**). IL-8 mRNA expression peaked at 3 h-treatment (**Figure 1C**) and IL-8 protein secretion was dose-dependently increased up to 24 h-treatment (**Figure 1D**).

### Lp.LTA Exclusively Inhibits Poly I:C-Induced IL-8 Production in IPEC-J2 Cells

**FIGURE 2:**
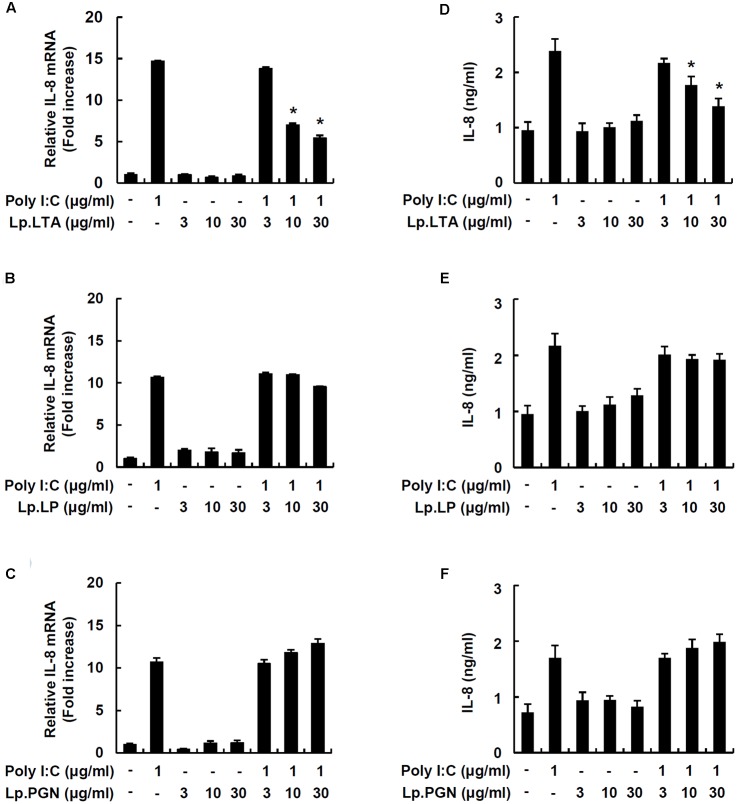
Lp.LTA inhibits poly I:C-induced IL-8 production in IPEC-J2 cells. IPEC-J2 cells (5 × 10^5^ cells/ml) were plated in 12-well culture plates and co-treated with poly I:C (1 μg/ml) and Lp.LTA (3–30 μg/ml) **(A)**, Lp.LP (3–30 μg/ml) **(B)**, or Lp.PGN (3–30 μg/ml) **(C)** for 3 h in serum-free medium. Then, total RNA was extracted and IL-8 mRNA expression was determined by real-time PCR. Data are mean ± standard deviation of triplicate samples. Cells were co-treated with poly I:C (1 μg/ml) and Lp.LTA (3–30 μg/ml) **(D)**, Lp.LP (3–30 μg/ml) **(E)**, or Lp.PGN (3–30 μg/ml) **(F)** for 24 h to examine the supernatant for IL-8 protein using ELISA. Data are mean ± standard deviation of triplicate samples. Asterisk (^∗^) indicates a significant difference compared with poly I:C-treated control group (*P* < 0.05).

In order to investigate whether major cell wall components were responsible for the inhibitory effect on porcine intestinal inflammatory responses, IPEC-J2 cells were treated with LTA, lipoproteins or PGN from *L. plantarum* in the presence of poly I:C. Only Lp.LTA inhibited IL-8 mRNA expression at 10 and 30 μg/ml. However, lipoproteins of *L. plantarum* (Lp.LP) or PGN of *L. plantarum* (Lp.PGN) did not inhibit poly I:C-induced IL-8 mRNA expression (**Figures 2A–C**). Similar to the results for mRNA, Lp.LTA, but not Lp.LP or Lp.PGN, significantly inhibited poly I:C-induced IL-8 secretion at 10 and 30 μg/ml (**Figures 2D–F**).

### *Lactobacillus* LTAs Do Not Commonly Inhibit Poly I:C-Induced IL-8 Production in IPEC-J2 Cells

**FIGURE 3:**
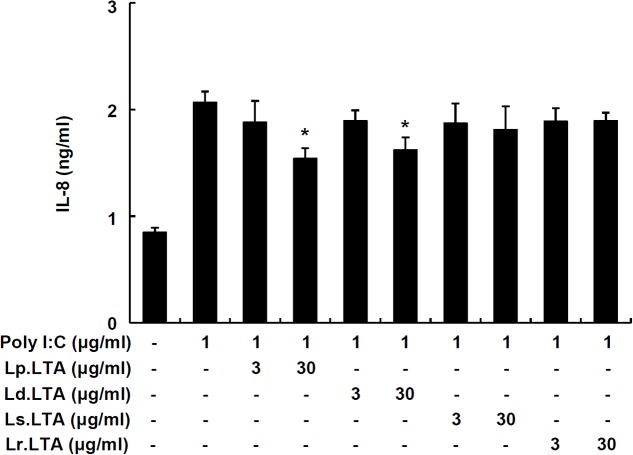
*Lactobacillus* LTAs do not commonly inhibit poly I:C-induced IL-8 production in IPEC-J2 cells. IPEC-J2 cells (5 × 10^5^ cells/ml) were plated in 96-well culture plates and treated with poly I:C (1 μg/ml) in the presence or absence of Lp.LTA, *L. delbrueckii* (Ld.LTA), *L. sakei* (Ls.LTA), or *L. rhamnosus* GG (Lr.LTA) at 3 or 30 μg/ml for 24 h in serum-free medium. IL-8 production in supernatants was determined by ELISA. Data are expressed as mean ± standard deviation of triplicate samples. Asterisk (^∗^) indicates significant difference compared with poly I:C-treated control group (*P* < 0.05).

To further examine whether other probiotic *Lactobacillus* LTAs inhibit poly I:C-induced IL-8 production, IPEC-J2 cells were treated with poly I:C and various *Lactobacillus* LTAs. **Figure 3** displayed that Lp.LTA and LTA from *L. delbrueckii* (Ld.LTA) significantly inhibited poly I:C-induced IL-8 secretion at 30 μg/ml. However, LTAs from *L. sakei* (Ls.LTA) and *L. rhamnosus* GG (Lr.LTA) did not inhibit poly I:C-induced IL-8 secretion, suggesting that other *Lactobacillus* LTAs did not commonly inhibit poly I:C-induced IL-8 production in IPEC-J2 cells.

### D-Alanine and Lipid Moieties of Lp.LTA Are Critical for Inhibition of Poly I:C-Induced IL-8 Production in IPEC-J2 Cells

**FIGURE 4:**
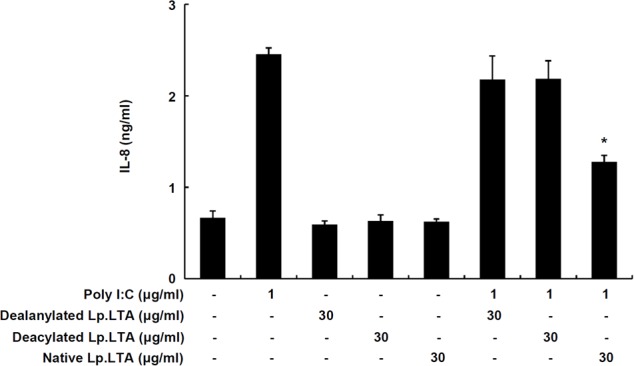
D-Alanine and lipid moieties of Lp.LTA are essential for inhibition of poly I:C-induced IL-8 production in IPEC-J2 cells. IPEC-J2 cells (5 × 10^5^ cells/ml) were plated in 96-well culture plates and treated with poly I:C (1 μg/ml) in the presence or absence of dealanyated Lp.LTA, deacylated Lp.LTA or native Lp.LTA (all 30 μg/ml) for 24 h. And then, IL-8 secretion in the supernatant was determined by ELISA. Data are expressed as mean ± standard deviation of triplicate samples. Asterisk (^∗^) indicates significant difference compared with appropriate control (*P* < 0.05).

In order to determine whether the D-alanine moiety of Lp.LTA was involved in the inhibition of poly I:C-induced IL-8 production, IPEC-J2 cells were treated with dealanylated Lp.LTA in the presence of poly I:C. Similar to results with native Lp.LTA, dealanylated Lp.LTA alone did not induce IL-8 secretion in IPEC-J2 cells. Although native Lp.LTA significantly inhibited poly I:C-induced IL-8 secretion, dealanylated Lp.LTA failed to inhibit poly I:C-induced IL-8 secretion (**Figure 4**). Next, we examined the inhibitory potential of deacylated Lp.LTA. Deacylated Lp.LTA did not inhibit poly I:C-induced IL-8 secretion (**Figure 4**). These results indicated that D-alanine and lipid moieties of Lp.LTA were involved in the inhibition of poly I:C-induced IL-8 production in IPEC-J2 cells.

### Lp. LTA Decreases Poly I:C-Induced MAPK Phosphorylation and NF-κB Activation

**FIGURE 5:**
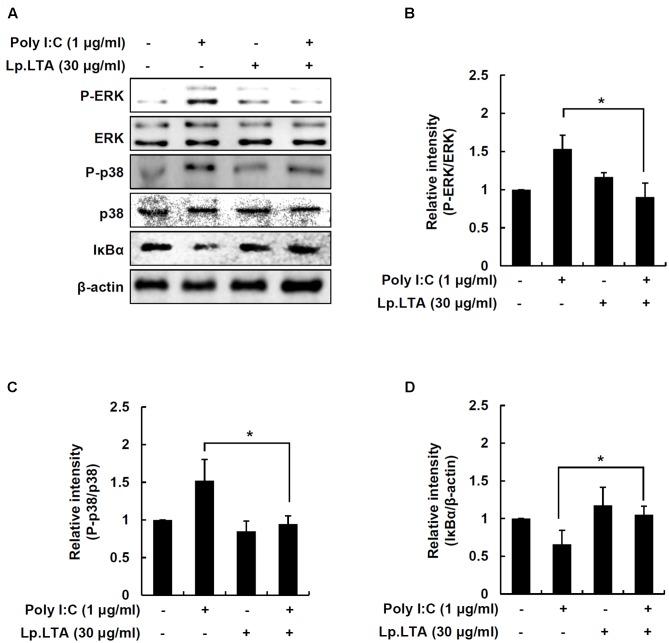
Lp.LTA reduces poly I:C-induced activation of p38 kinase, ERK and NF-κB in IPEC-J2 cells. IPEC-J2 cells (5 × 10^5^ cells/ml) were grown in serum-free medium overnight. Cells were treated with poly I:C (1 μg/ml) in the presence or absence of Lp.LTA (30 μg/ml) for 30 min. Cells were lysed and equal amounts of proteins were analyzed by Western blots using antibodies specific to phosphorylated or non-phosphorylated ERK, p38 kinase or IκBα **(A)**. The intensity of each band was determined using an imaging densitometer and relative protein level calculated based on intensity of non-phosphorylated ERK **(B)**, non-phosphorylated p38 **(C)**, or β-actin **(D)**. Data are expressed as mean ± standard deviation from three independent experiments. Asterisk (^∗^) indicates significant difference compared with appropriate control (*P* < 0.05).

IPEC-J2 cells were treated with poly I:C and/or Lp.LTA to determine MAPK phosphorylation and NF-κB activation. Poly I:C alone enhanced ERK and p38 phosphorylation, but the presence of Lp.LTA significantly decreased poly I:C-mediated phosphorylation of ERK and p38 (**Figures 5A–C**). Moreover, poly I:C alone induced IκBα degradation, leading to NF-κB activation, whereas Lp.LTA blocked poly I:C-induced IκBα degradation, suggesting that Lp.LTA reduced NF-κB activation (**Figures 5A,D**). It is likely that Lp.LTA regulated MAPK phosphorylation and NF-κB activation, leading to the reduction of poly I:C-induced IL-8 production in IPEC-J2.

## Discussion

In the livestock industry, probiotics are used in piglets to improve growth and digestibility, prevent diarrhea, and modulate immune responses in the gut (Hou et al., 2015). A number of pathogenic bacteria and several viruses including group A rotavirus are associated with acute diarrhea in weaning and post-weaning piglets (Martella et al., 2010). Although the use of probiotics is widely accepted as an alternative to antibiotics, the effector molecules of probiotic bacteria responsible for the prevention of virus-induced diarrhea in piglets have not been identified. In this study, we demonstrated that a major cell wall component, LTA of probiotic *L. plantarum*, inhibited a synthetic analog of double-stranded RNA derived from viruses, poly I:C-induced IL-8 production in porcine intestinal epithelial cells.

Unlike LTA from pathogenic bacteria, LTA from probiotic bacteria such as *Lactobacillus* distinctively acts as an anti-inflammatory substance. For example, Lp.LTA suppressed a synthetic lipopeptide, Pam2CSK4-induced IL-8 production in human intestinal epithelial cells (Noh et al., 2015). LPS-induced endotoxin shock was reduced by the administration with Lp.LTA in a mouse model, which up-regulated IL-1R-associated kinase-M, a negative regulator, to lessen excessive inflammatory responses (Kim et al., 2008). In accordance with previous observations, Lp.LTA effectively inhibited TLR3-mediated inflammatory responses in porcine intestinal epithelial cells. It is likely that Lp.LTA may have broad spectrum of inhibitory actions on bacterial and viral inflammatory responses. In addition, D-alanine and lipid moieties of LTA are involved in immunostimulating potential (Ryu et al., 2009). Deficiency of D-alanine or lipid moieties of Lp.LTA completely lost the inhibitory effect on IL-8 production, suggesting that D-alanine and lipid moieties of Lp.LTA are essential components in the inhibition of poly I:C-induced IL-8 production in porcine intestinal epithelial cells.

TLR2 is known to associate with the pathogenesis of microbial infections (Kang et al., 2016). It is also important for host protection against infection. For instance, Lp.LTA attenuated MAPK phosphorylation and NF-κB activation by reducing TLR2 activation, resulting in an anti-inflammatory response in human intestinal epithelial cells (Noh et al., 2015). This observation is similar to our study, in which Lp.LTA as a TLR2 ligand regulated MAPK phosphorylation and NF-κB activation, presuming that interaction of Lp.LTA with TLR2 was involved in the anti-inflammatory response. In contrast to our finding, TLR2 combined with TLR3 enhances the induction of inflammatory cytokines such as tumor necrosis factor (TNF)-α and IL-6, but inhibits IFN-stimulated genes such as IFN-β in myeloid dendritic cells (Vanhoutte et al., 2008). However, among TLR2 ligands such as LTA, PGN, and synthetic lipopeptides, LTA from *Staphylococcus epidermis* and *S. aureus* suppressed poly I:C-induced TNF-α, blocking the activation of NF-κB by up-regulating the negative regulatory molecule TNF receptor-associated factor-1 (Lai et al., 2009). These results suggest that a negative regulatory mechanism(s) might be involved in the inhibitory effect of Lp.LTA on IL-8 production in porcine intestinal epithelial cells.

Growing evidence supports the therapeutic and preventive applications of probiotics against gastrointestinal inflammatory reactivity in animals and humans. Of note, probiotic effects are often species- or strain-specific (Lee et al., 2013). We demonstrated that Lp.LTA contributed to the anti-inflammatory responses in porcine intestines. Our results provide an understanding of the interaction between a probiotic effector molecule and intestinal epithelial cells for the prevention or treatment of gut inflammatory disorders in the livestock. This study clearly showed that LTA as a probiotic effector molecule contributed to the probiotic interaction with porcine intestinal epithelial cells that is feasible to be used in animal feeding.

## Author Contributions

S-SK, C-HY, and SHH conceived the research. KWK, S-JW, and S-SK performed the experiments. S-SK, C-HY, and SHH wrote the manuscript. O-JP, K-DS, KBA, and H-KL interpreted data and edited manuscript. All authors reviewed and accepted the manuscript.

## Conflict of Interest Statement

The authors declare that the research was conducted in the absence of any commercial or financial relationships that could be construed as a potential conflict of interest.
